# Loss function of SL (sekiguchi lesion) in the rice cultivar Minghui 86 leads to enhanced resistance to (hemi)biotrophic pathogens

**DOI:** 10.1186/s12870-020-02724-6

**Published:** 2020-11-04

**Authors:** Dagang Tian, Fang Yang, Yuqing Niu, Yan Lin, Zaijie Chen, Gang Li, Qiong Luo, Feng Wang, Mo Wang

**Affiliations:** 1 Biotechnology Research Institute, Fujian Key Laboratory of Genetic Engineering for Agriculture, Fujian Academy of Agricultural Sciences, Fuzhou, 350003 Fujian China; 2 State Key Laboratory of Ecological Pest Control for Fujian and Taiwan Crops, College of Life Science, Fujian Agriculture and Forestry University, Fuzhou, 350002 Fujian China; 3 Key Laboratory of Ministry of Education for Genetics, Breeding and Multiple Utilization of Crops, College of Agriculture, Fujian Agriculture and Forestry University, Fuzhou, 350002 Fujian China; 4 Ministry of Education Key Laboratory of Agriculture Biodiversity for Plant Disease Management, Yunnan Agricultural University, Kunming, 650201 China; 5 Fujian University Key Laboratory for Plant–Microbe Interaction, Fujian Agriculture and Forestry University, Fuzhou, 350002 Fujian China

**Keywords:** Rice, Serotonin, Reactive oxygen species, PAMP-triggered immunity, Defense hormones, *Pyricularia oryzae*

## Abstract

**Background:**

Serotonin, originally identified as a neurotransmitter in mammals, functions as an antioxidant to scavenge cellular ROS in plants. In rice, the conversion of tryptamine to serotonin is catalyzed by SL (sekiguchi lesion), a member of cytochrome P450 monooxygenase family. The *sl* mutant, originated from rice cultivar Sekiguchi-asahi, exhibits spontaneous lesions, whereas its immune responses to pathogens have not been clearly characterized.

**Results:**

Here we identified three allelic mutants of *SL* in an *indica* rice restore line Minghui 86 (MH86), named as *sl-MH-1*, *− 2* and *− 3*, all of which present the typical lesions under normal growth condition. Compared with those in MH86, the serotonin content in *sl-MH-1* is dramatically decreased, whereas the levels of tryptamine and L-trytophan are significantly increased. The *sl-MH-1* mutant accumulates high H_2_O_2_ level at its lesion sites and is more sensitive to exogenous H_2_O_2_ treatment than the wild type. When treated with the reductant vitamin C (Vc), the lesion formation on *sl-MH-1* leaves could be efficiently suppressed. In addition, *sl-MH-1* displayed more resistant to both the blast fungus and blight bacteria, *Pyricularia oryzae* (*P. oryzae*, teleomorph: *Magnaporthe oryzae*) and *Xanthomonas oryzae* pv. *Oryzae* (*Xoo*), respectively. The pathogen-associated molecular patterns (PAMPs)-triggered immunity (PTI) responses, like reactive oxygen species (ROS) burst and callose deposition, were enhanced in *sl-MH-1*. Moreover, loss function of SL resulted in higher resting levels of the defense hormones, salicylic acid and jasmonic acid. The RNA-seq analysis indicated that after *P. oryzae* infection, transcription of the genes involved in reduction-oxidation regulation was the most markedly changed in *sl-MH-1*, compared with MH86.

**Conclusions:**

Our results indicate that SL, involving in the final step of serotonin biosynthesis, negatively regulates rice resistance against (hemi)biotrophic pathogens via compromising the PTI responses and defense hormones accumulation.

**Supplementary Information:**

The online version contains supplementary material available at 10.1186/s12870-020-02724-6.

## Background

In nature, plants are constantly exposed to a wide range of pathogenic microorganisms, thus they have developed a sophisticated innate immune system to protect themselves from infection [1]. PAMP-triggered immunity (PTI), activated upon recognition of conserved pathogen-associated molecular patterns (PAMPs) by plant cell membrane-localized pattern recognition receptors, can rapidly elicit plant defense responses, which includes calcium ions influx, reactive oxygen species (ROS) generation, callose deposition and stomatal closure et al [2]. Another branch of plant immunity is effector-triggered immunity (ETI), caused by direct or indirect perception of pathogen effectors via plant resistance (R) proteins, which is often associated with a hypersensitive response (HR) and renders plants the isolate-specific resistance [3]. As a typical feature of HR, programmed cell death (PCD), accompanied with production of ROS and phytoalexins, is employed to effectively halt the spread of pathogens within the initial penetration site [4]. On the other hand, activation of plant immune responses depends on dramatic changes in the cellular reduction-oxidation (redox) status, resulting in the reprogramming of the transcriptome and the establishment of both local and systemic defense [5]. Upon being infected, the raised oxidative stress in plant cells is essential for HR formation and development [6].

Serotonin (5-hydroxytryptamine) is originally known as a neurotransmitter controlling fundamental physiological processes, such as mood, sleep and anxiety, in mammals [7]. Since its first identification in the *Mucuna pruriens*, serotonin has been found widely distributed in the plant kingdom and involved in regulation of diverse physiological processes [8, 9]. Serotonin biosynthesis in plants occurs as that tryptophan is converted into tryptamine by tryptophan decarboxylase, following with the catalysis of tryptamine to serotonin by tryptamine 5-hydroxylase [10]. Serotonin is readily oxidized and functions as an antioxidant to scavenge cellular ROS [11]. And the antioxidant activity of serotonin is reported to far exceed that of tryptophan, tryptamine and its derivatives [9]. Thus, it is conceivable that serotonin plays a role in plant innate immunity via regulating cellular redox status. Moreover, serotonin could also be incorporated into the plant cell wall for strengthening the mechanical barrier against pathogens [12].

Rice (*Oryza sativa*) is the staple food for more than half of the world’s population. Rice blast and bacterial blight, caused by the hemibiotrophic fungal pathogen *Pyricularia oryzae* (*P. oryzae*) and the biotrophic bacterial pathogen *Xanthomonas oryzae* pv. *Oryzae* (*Xoo*), respectively, are the most devastating rice diseases [13]. Increasing reports indicate that the serotonin pathway in rice is involved in disease resistance, and most of studies are based on characterizing a lesion mimic mutant, *sekiguchi lesion* (*sl*), originated from the rice cultivar Sekiguchi-asahi. The *SL* gene, cloned by Fujiwara *et. al* in 2010, encodes CYP71P1, belonging to the cytochrome P450 monooxygenase family [14]. SL possesses tryptamine 5-hydroxylase enzyme activity and can catalyze the conversion of tryptamine to serotonin in rice. As this reason, the accumulation of serotonin after *Bipolaris oryzae* (*B. oryzae*, a necrotrophic pathogen) infection was abolished in *sl* mutant, and *sl* displayed increased susceptibility to *B. oryzae* [12]. However, the responses of *sl* mutant to biotrophic pathogens infection and the mechanisms of SL involved in plant immunity are still ambiguous.

In this study, we identified the *sl* mutants in MH86 background (*sl-MH*) and found that the lesions of *sl-MH-1* is caused by excessive accumulation of ROS, which could be suppressed by the reductant treatment. The absence of SL results in the enhanced PTI responses and high resting levels of defense hormones, and thus the broad-spectrum resistance against *P. oryzae* and *Xoo*.

## Results

### Identification of the *sl* mutants in MH86 background

We have identified a rice runaway-cell death mutant, *rcd1–1*, in an *indica* rice restore line Minghui 86 (MH86). This mutant spontaneously exhibits orange-colored lesions on its leaves when grown in field or greenhouse (Fig. S1). Through map-based cloning, a G to T mutation was found at 1205 nucleotide of *SL* ORF, which leads to the 370 Arg mutated to Leu [15]. Two allelic mutants, *rcd1–2* and *rcd1–3*, were obtained by ^60^Co ~ γ-ray radiation, which carries C85 and A1420 deletion in *SL* coding region, respectively (Fig. S2). Both *rcd1–2* and *rcd1–3* spontaneously present sekiguchi lesions, similar with *rcd1–1* (Fig. S1). Therefore, hereafter we named *rcd1–1*, *rcd1–2* and *rcd1–3* mutants as *sl-MH-1*, *sl-MH-2* and *sl-MH-3*, respectively.

**Fig. 1:**
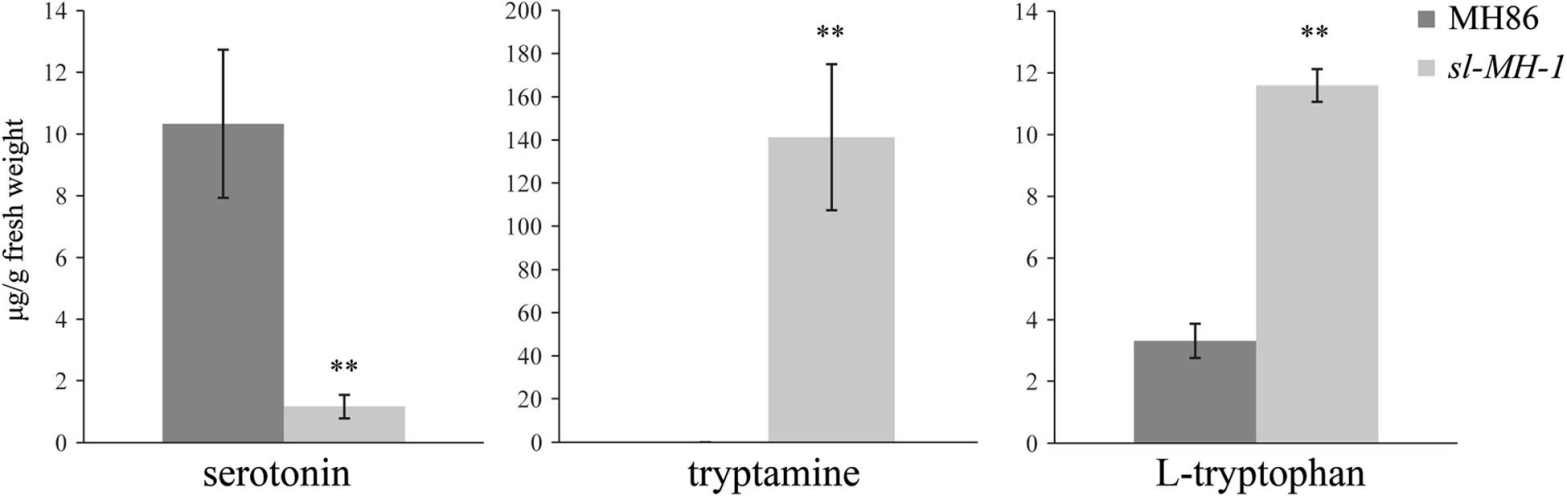
Levels of serotonin, tryptamine and L-tryptophan in MH86 and *sl-MH-1*. The contents of serotonin, tryptamine and L-tryptophan in the leaves of 8-week-old MH86 and *sl-MH-1* plants were measured by Ultra High Performance Liquid Chromatography (UHPLC). Bars represent mean values ± standard error (SE) from five biological replicates. Statistically significant difference was indicated by ** (*p* < 0.01, Student’s *t*-test)

To investigate difference of the metabolites levels in serotonin biosynthesis pathway in *sl-MH-1* mutant and MH86, the contents of serotonin, tryptamine and L-trytophan from the leaves of 8-week-old plants grown in a greenhouse with natural light were measured. The data indicated that serotonin level was significantly decreased in *sl-MH-1* compared with MH86, whereas tryptamine and L-trytophan, the upstream metabolites in serotonin biosynthesis, were accumulated to significantly higher levels in *sl-MH-1* (Fig. 1), proving the function of SL in catalyzing the conversion of tryptamine to serotonin. Moreover, we found that the content of L-glutamine in *sl-MH-1* was significantly higher than that in MH86 (Fig. S3). Taken together, our results indicate that mutation of *SL* in MH86 background also results in the typical sekiguchi lesion and compromised serotonin biosynthesis.

### Endogenous oxidative stress results in lesion formation on *sl-MH-1* leaves

**Fig. 2:**
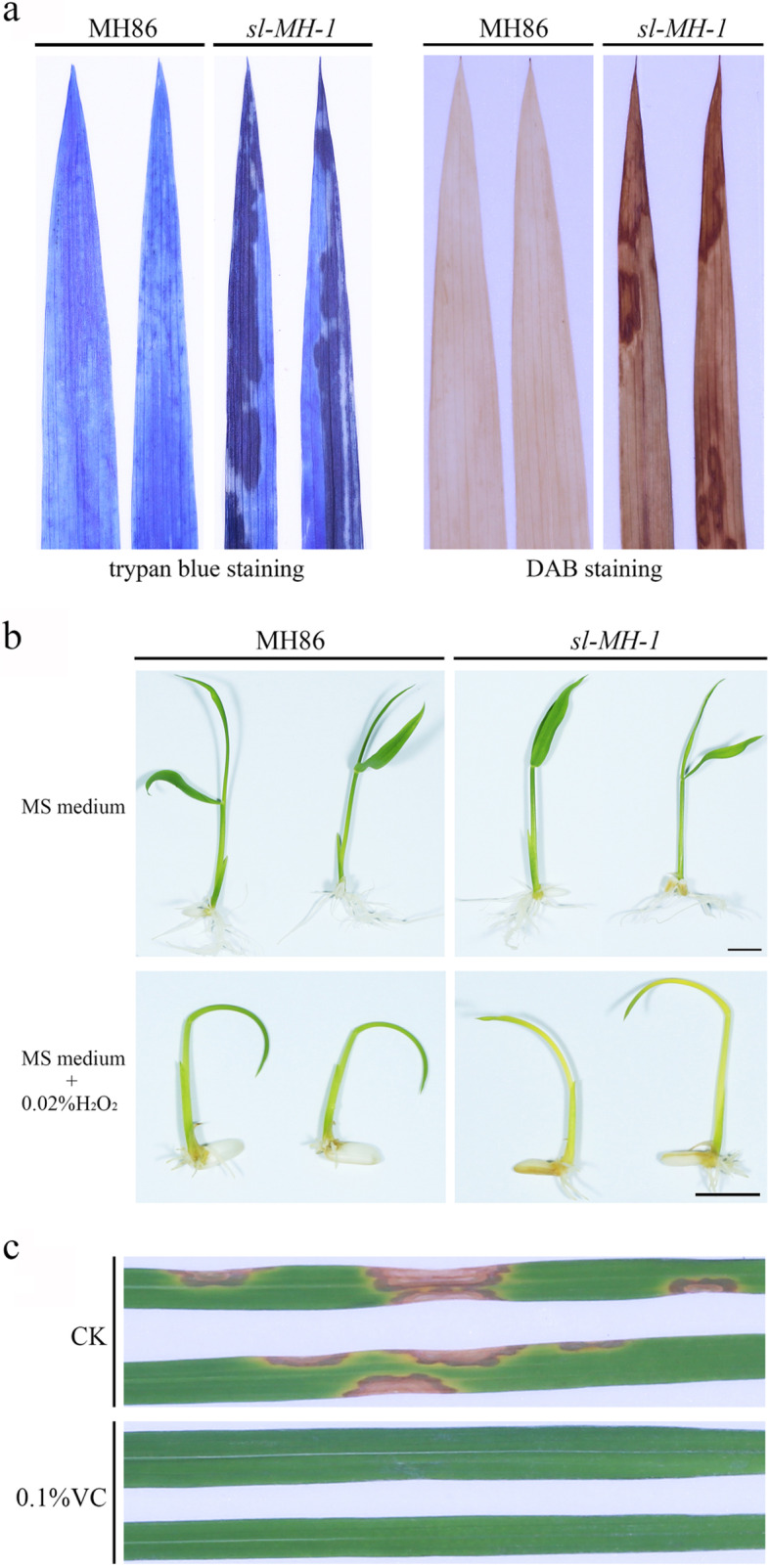
Responses of *sl-MH-1* to exogenous H_2_O_2_ and VC treatments. **a** Trypan blue (left) and DAB (right) staining of the 8-week-old *sl-MH-1* and MH86 leaves to detect cell death and H_2_O_2_ accumulation, respectively. **b** *sl-MH-1* and MH86 seeds were germinated on MS medium with or without 0.02% H_2_O_2_. The seedlings were imaged after kept in a growth chamber for 7 days. Bars = 1 cm. **c**. The leaves of *sl-MH-1* plants were imaged after treated with 0.1% VC for 10 days. CK means water treatment as control

To determine the relationship between cell death and ROS accumulation in absence of SL, we stained the leaves of 8-week-old *sl-MH-1* and MH86 with trypan blue and 3,3-diaminobenzidine tetrahydrochloride (DAB) to detect cell death and H_2_O_2_ accumulation, respectively. As shown in Fig. 2a, compared with MH86, the lesion sites on *sl-MH-1* leaves (shown by the dark trypan blue staining) displayed more H_2_O_2_ accumulation as indicated by DAB staining (dark brown color). To assess the defects of *sl-MH-1* responding to exogenous ROS stress, *sl-MH-1* and MH86 seeds were germinated on Murashige and Skoog (MS) medium with or without H_2_O_2_. After kept in a 28 °C growth chamber with 12-h light for 1 week, we found that there was no significant difference between *sl-MH-1* and MH86 seedlings on MS medium without H_2_O_2_ (Fig. 2b). The H_2_O_2_ application could retard the growth of both *sl-MH-1* and MH86 seedlings, whereas *sl-MH-1* seedlings displayed more stressed (with leaves turning yellow) than the wild type to H_2_O_2_ treatment (Fig. 2b). Therefore, *SL* mutation leads to high endogenous ROS accumulation and increased sensitivity to exogenous ROS stress.

In order to further determine whether lesion of *sl-MH-1* is resulted from its high internal ROS accumulation, we treated the mutant with the antioxidant Vitamin C (VC, ascorbic acid), which is one of the major redox buffers in plant cell and functions as an antioxidant to conduct ROS detoxification [16]. And it has been reported that exogenous application of VC could alleviate oxidative stress in rice induced by abiotic stress [17]. 3-week-old *sl-MH-1* seedlings grown in green house, whose leaves did not exhibit visible lesions, were treated with 0.1% VC or water (as control) by spraying for 10 days. Then the lesion numbers on the top second and third leaves were counted. As shown in Fig. 2c and Table 1, the lesion numbers on *sl-MH-1* leaves was significantly decreased by VC treatment, indicating that exogenous antioxidant application could suppress *sl-MH-1* lesion formation. Taken together, our data suggest that the cell death in absence of SL is caused by high endogenous ROS accumulation.

**Table 1:** Lesion numbers on the *sl-MH-1* leaves after VC and CK treatments

treatment	average number of lesions per leaf	
	top second leaf	top third leaf
CK (86 plants)	1.33 + 1.38	1.24 + 1.41
0.1%VC (42 plants)	0.05 + 0.22**	0.70 + 0.26**

** means statistically significant with *p* < 0.01 with One-way analysis of variance (ANOVA)

### *sl-MH-1* mutant displays enhanced resistance to *P. oryzae* and *Xoo*

**Fig. 3:**
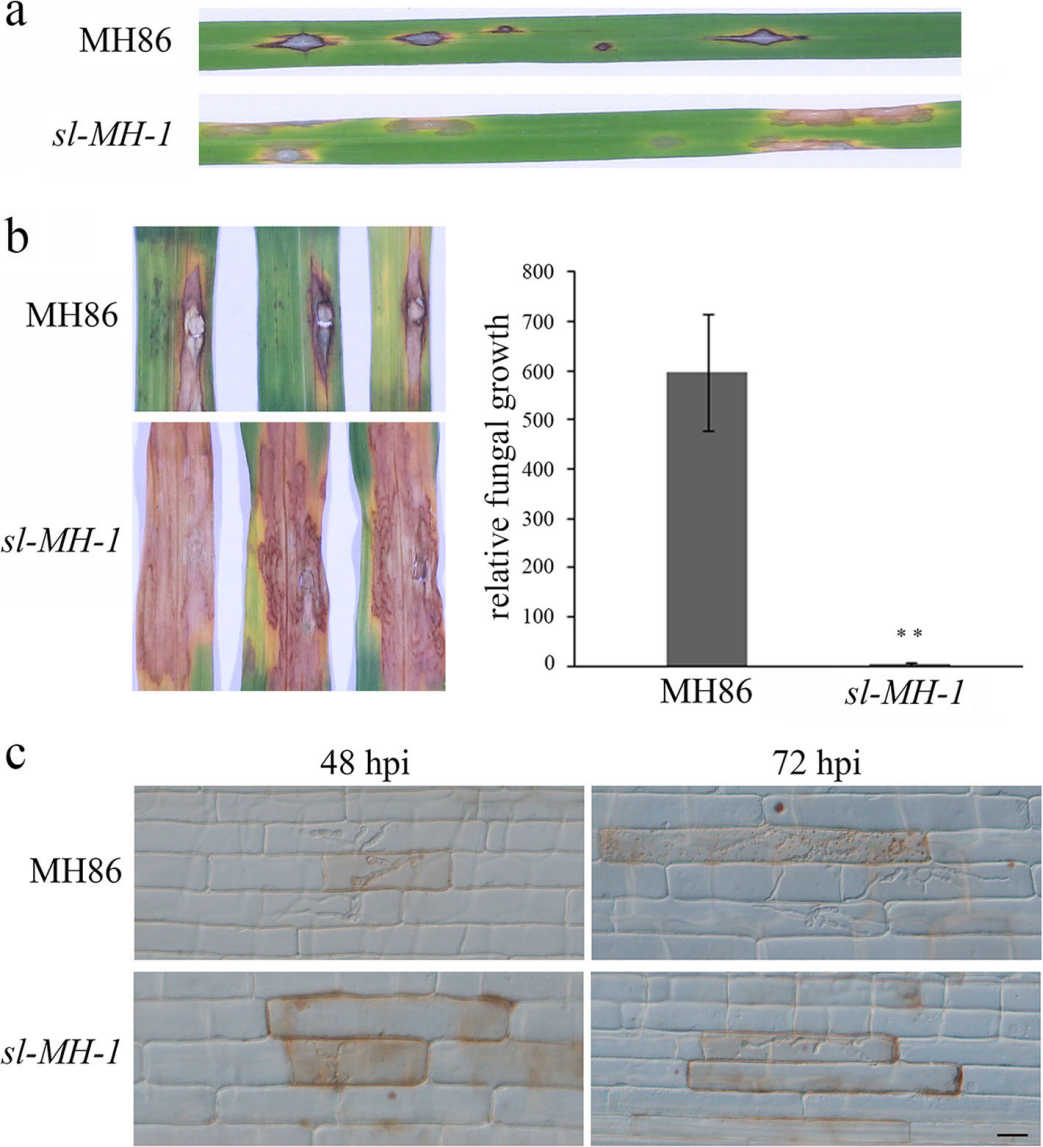
*sl-MH-1* mutant displays increased resistance to *P. oryzae*. **a** 3-week-old MH86 and *sl-MH-1* seedlings were inoculated with *P. oryzae* conidia by spraying. At 7 days post inoculation (dpi), the diseased leaves were imaged. **b** Punch inoculation with *P. oryzae* conidia was carried out on the leaves of 4-week-old MH86 and *sl-MH-1* plants. The infected leaves were photographed at 9 dpi (left); meanwhile fungal biomass was measured to quantify the relative *P. oryzae* growth in MH86 and *sl-MH-1* leaves (right). Bars represent mean values ± SD from three biological replicates. Statistically significant difference was indicated by ** (*p* < 0.01, Student’s *t*-test). **c**. Leaf sheathes of MH86 and *sl-MH-1* were inoculated with *P. oryzae* conidia to monitor the infection process. The infected cells were imaged at 48 and 72 hpi with a microscope under bright field. Bar = 20 μm

As ROS production is an important defense response of plants to pathogen invasion, we wondered whether *sl-MH-1* mutant was more resistant to rice pathogens. Firstly, we challenged 3-week-old *sl-MH-1* and MH86 seedlings with conidial spores of the compatible blast fungal isolate, FJ-1, by spraying. As shown in Fig. 3a, inoculation with FJ-1 caused typical disease symptom on MH86 leaf, whereas *sl-MH-1* mutant exhibited sekiguchi lesions at the FJ-1 infection sites. To quantify the resistance of *sl-MH-1* to the blast fungus, we carried out punch inoculation on *sl-MH-1* and MH86 leaves and investigated the relative fungal biomass within the infected region, which showed that the *sl-MH-1* mutant supported significantly less blast fungus growth than MH86 (Fig. 3b). In order to further monitor the process of *M. oryzae* invasion in *sl-MH-1* and MH86, a rice leaf sheath inoculation assay was performed. It was found that the invasive hyphae (IH) in MH86 could extend to the neighboring cells of the first infected cell at 48 h post inoculation (hpi) and further extended to the adjacent cells at 72 hpi (Fig. 3c). However, at both 48 and 72 hpi, the IH was still constricted in the first infected cell of *sl-MH-1* (Fig. 3c). Although the IH could elongate from 48 to 72 hpi in *sl-MH-1* cells, the infection rate of blast fungus was markedly slower than that in MH86. Taken together, these results indicate that knockout of *SL* enhances rice resistance to blast fungus.

**Fig. 4:**
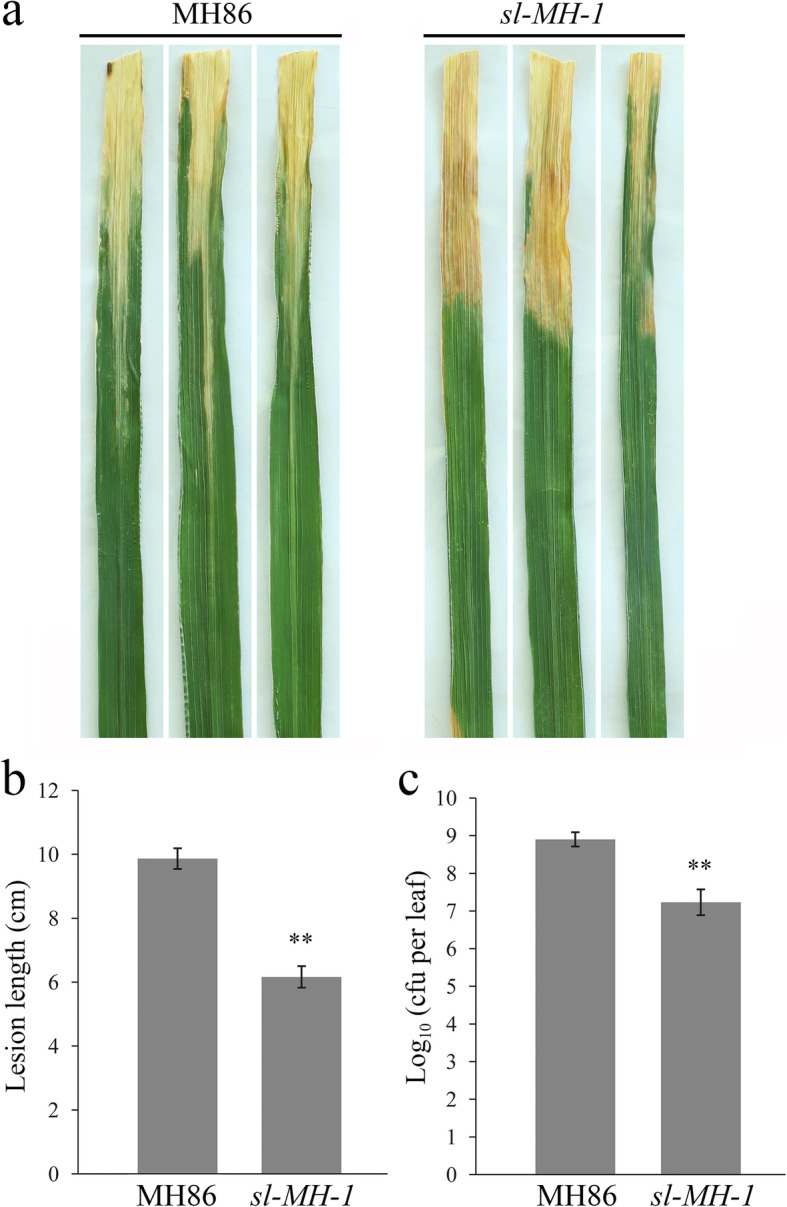
*sl-MH-1* mutant showed enhanced resistance to *Xoo*. **a** MH86 and *sl-MH-1* plants were inoculated with *Xoo* at the tilling stage. The infected leaves from three independent MH86 or *sl-MH-1* plants were imaged at 14 dpi. **b** Blight lesion length on *sl-MH-1* and MH86 leaves were measured. Bars represent mean values ± SD (*n* = 6, from three independent plants). **c** Blight bacterial populations were counted 14 dpi with bars representing mean values ± SD from three independent MH86 or *sl-MH-1* plants. Cfu means colony-forming units. Statistically significant difference in b and c was indicated by ** (*p* < 0.01, Student’s *t*-test)

To determine whether *sl-MH-1* mutant possesses the increased resistance to bacterial pathogens, we further inoculated *sl-MH-1* and MH86 plants with *Xoo*. As shown in Fig. 4, via comparing blight lesion length and bacterial population at 14 days after inoculation, it was found that *sl-MH-1* indeed displayed enhanced resistance to blight bacteria as well. Furthermore, to support the resistant phenotypes of *sl-MH-1* is resulted from loss function of SL, another allelic mutant, *sl-MH-3*, was subjected to the above inoculation assays. The results showed that similar with *sl-MH-1*, *sl-MH-3* displayed more resistance to *P. oryzae* and *Xoo* (Fig. S4a-d). Therefore, SL negatively regulates rice broad-spectrum resistance to the (hemi)biotrophic pathogens.

### *SL* mutation leads to enhanced PTI responses

**Fig. 5:**
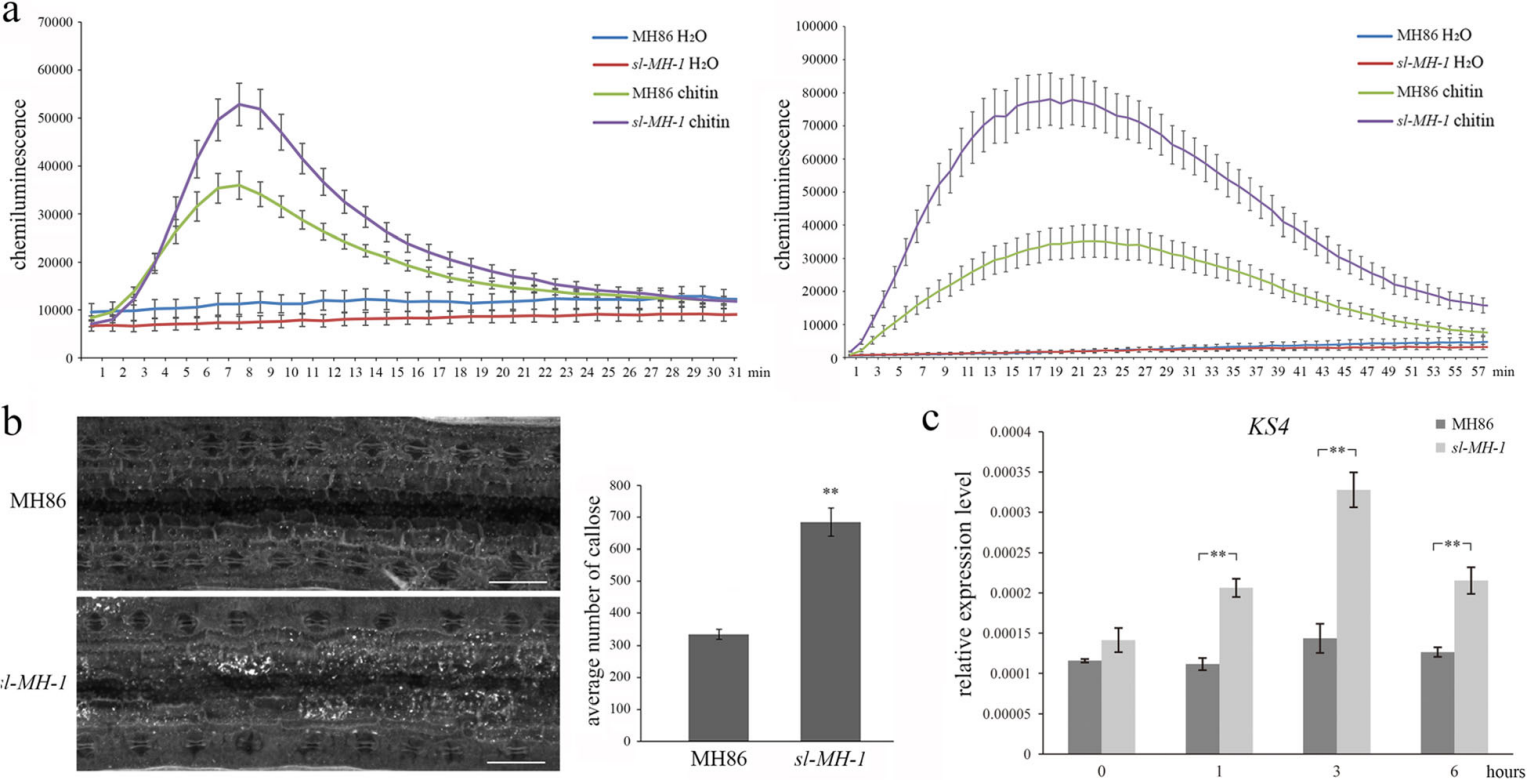
PTI responses are enhanced in *sl-MH-1* mutant. **a** ROS burst from MH86 and *sl-MH-1* leaf discs treated with 400 nM chitin or 500 nM flg22 was detected at the indicated time points. Error bars represent the SE (*n* = 8). The data are from one of three independent experiments performed with similar results. B Callose deposition on MH86 and *sl-MH-1* leaves after chitin treatment was imaged with a microscope under UV light (left). Number of the callose deposition per view was analysed by Image J (right). Bars represent mean values ± SE (*n* = 5). Statistically significant difference was indicated by ** (*p* < 0.01, Student’s *t*-test). This assay was performed in three independent replicates with similar results. **c** The relative transcriptional levels of *KS4* in 3-week-old MH86 and *sl-MH-1* leaves at the indicated time points after treated with 400 nM chitin. Rice ubiquitin-encoding gene (*UBQ*) was used as the internal control. Bars represent mean values ± SD (*n* = 3). Statistically significant difference was indicated by ** (*p* < 0.01, Student’s *t*-test). This assay was performed in two independent replicates with similar results

In order to decipher the mechanism of enhanced resistance by SL deletion, the PTI responses of *sl-MH-1* and MH86 were analyzed. We firstly measured the levels of ROS burst in *sl-MH-1* and MH86 upon flg22 and chitin treatments. It was found that ROS burst could be stimulated in both *sl-MH-1* and MH86 leaves by the PAMPs treatments, whereas the levels of ROS production were significantly higher in *sl-MH-1* than MH86 at the indicated time points (Fig. 5a). Additionally, the callose deposition on *sl-MH-1* and MH86 leaves after chitin treatment was investigated. As shown in Fig. 5b, compared with MH86, there was significantly more callose deposited on *sl-MH-1* leaves. And the enhanced callose deposition was similarly observed on *sl-MH-3* leaves (Fig. S4e). Furthermore, transcriptional profile of the PTI-related defense gene *KS4* was investigated in *sl-MH-1* and MH86 before and after chitin treatment. KS4 is a diterpene cyclase enzyme involving in momilactone biosynthesis [18, 19]. We found that at all the indicated time points after chitin treatment, the transcriptional levels of *KS4* in *sl-MH-1* were significantly higher than those in MH86 (Fig. 5c). Taken together, our data demonstrate that loss function of SL enhances rice PTI responses.

### *sl-MH* mutants accumulate higher resting levels of defense hormones

**Fig. 6:**
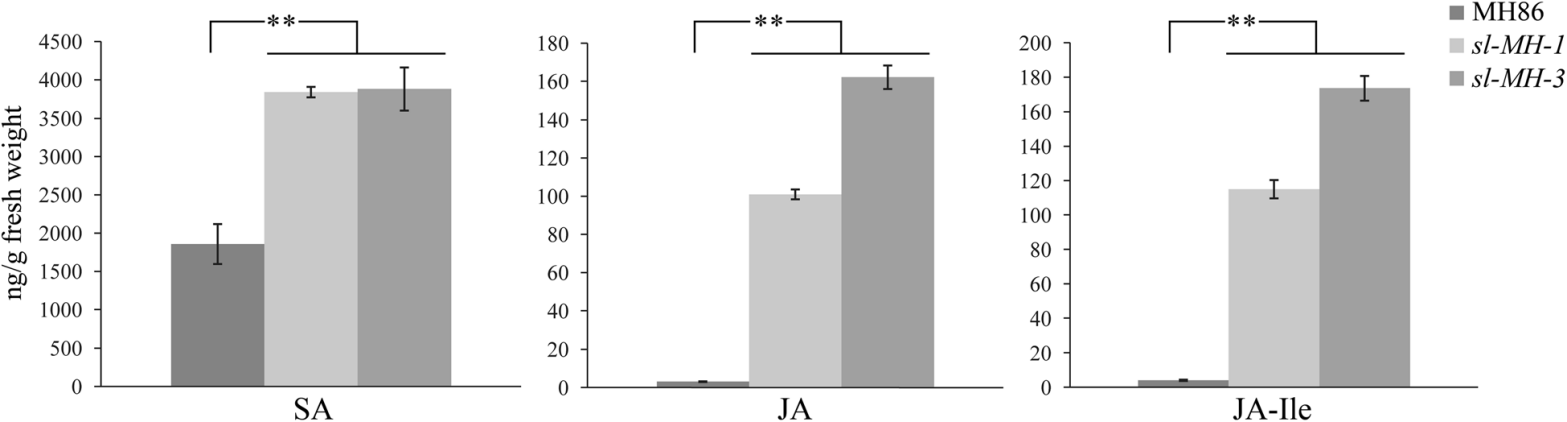
Contents of SA, JA and JA-Ile in MH86 and *sl-MH* mutants. The resting levels of SA, JA and JA-Ile in 6-week-old MH86, *sl-MH-1* and *sl-MH-3* plants were measured by UPLC. Bars represent mean values ± SD from three biological replicates. Statistically significant difference was indicated by ** (*p* < 0.01, Student’s *t*-test)

Salicylic acid (SA) and jasmonic acid (JA) are the major phytohormones involved in defense against pathogens. To assess whether *SL* mutation alters contents of these defense hormones, we detected the levels of free SA, JA and JA-isoleucine (JA-Ile, the active form of JA) in 6 weeks-old *sl-MH-1* and MH86 plants, when the lesions were fully appeared on *sl-MH-1* leaves. As shown in Fig. 6, the free SA level in *sl-MH-1* was about twice as high as that in MH86; both JA and JA-Ile contents in *sl-MH-1* were burst to approximately 30 times higher than those in MH86. Meanwhile, levels of SA, JA and JA-Ile were also measured in *sl-MH-3* to confirm that the high accumulation of the defense hormones was resulted from *SL* mutation. It was found that *sl-MH-3* accumulated similar level of free SA with *sl-MH-1* and even higher levels of JA and JA-Ile than those in *sl-MH-1* (Fig. 6). Moreover, the levels of defense hormones were also measured in 3 weeks-old *sl-MH-1* and MH86 plants, when the lesions were not observed on *sl-MH-1* leaves. As shown in Fig. S5, the free SA level in 3-week-old *sl-MH-1* seedlings was similarly about two-fold higher than that in MH86; JA-Ile content in *sl-MH-1* was about 3 times higher than that in MH86, whereas no significant difference of JA level was detected between 3-week-old *sl-MH-1* and MH86. These results suggest that *SL* mutation leads to constantly more SA production and dramatically increased JA and JA-Ile contents along with the lesion appearance.

### *SL* mutation mainly alters transcription of the genes in reduction-oxidation pathway upon blast fungus infection

**Fig. 7:**
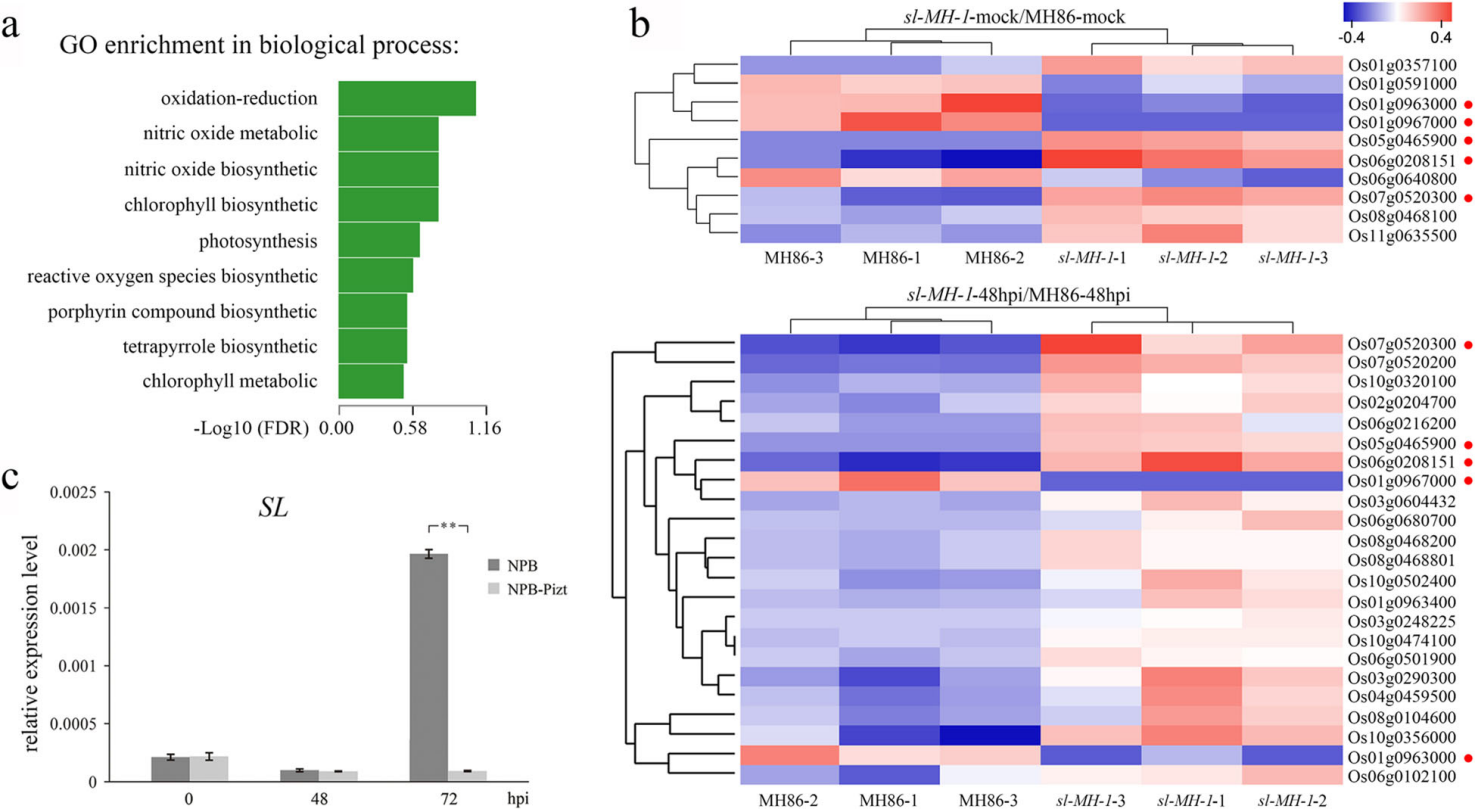
Transcritome analysis in MH86 and *sl-MH-1* plants with and without blast fungus inoculation. **a** Gene Ontology enrichment analysis showed the changed transcript profiles in various biological processes of *sl-MH-1*, compared with MH86, after *P. oryzae* inoculation. **b** Heat maps of the significant differentially expressed genes (DEGs) in the redox pathway from *sl-MH-1*-mock compared with MH86-mock and *sl-MH-1*-48hpi compared with MH86-48hpi. The red dots indicate the genes with similar trend of transcriptional change in *sl-MH-1*-mock/MH86-mock and *sl-MH-1*-48hpi/MH86-48hpi. **c** The relative transcriptional levels of *SL* in NPB and NPB-Pizt plants before and after inoculation with *P. oryzae* isolate KJ201. *UBQ* was used as the internal control. Bars represent mean values ± SD (*n* = 3). Statistically significant difference was indicated by ** (*p* < 0.01, Student’s *t*-test). The data are from one of three independent experiments performed with similar results

To determine the difference of gene transcription in *sl* mutant and wild type in response to pathogen infection, we carried out transcritome analysis in *sl-MH-1* and MH86 plants with and without *P. oryzae* inoculation. The leaf tissues of *sl-MH-1* and MH86 were harvested at 48 hpi with FJ-1 to conduct RNA-seq analysis, water treatment was used as control (mock). The number of significant differentially expressed loci (SDEL) in *sl-MH-1*-mock compared to MH86-mock (*sl-MH-1*-mock/MH86-mock) and *sl-MH-1*-48hpi compared to MH86-48hpi (*sl-MH-1*-48hpi/MH86-48hpi) with fold change ≥2 or ≤ 0.5 were 95 and 149, respectively (supplementary Table S1 and Table S2). We used Gene Ontology enrichment analysis to investigate the transcript profiles in various biological processes of *sl-MH-1* and MH86 following blast fungus infection. The results indicated that compared with MH86, the most markedly affected biological process in *sl-MH-1* is the redox pathway (Fig. 7a). The significant differentially expressed genes (DEGs) related to redox regualtion were 10 and 23 in *sl-MH-1*-mock/MH86-mock and *sl-MH-1*-48hpi/MH86-48hpi, respectively, whose transcript levels were displayed as heatmaps shown in Fig. 7b. There are five redox-related genes had similar trend of transcriptional change in both *sl-MH-1*-mock/MH86-mock and *sl-MH-1*-48hpi/MH86-48hpi (Fig. 7b, indicated by red dots), suggesting their transcriptional changes caused by defective SL was not affected by blast fungus infection. Among them, Os07g0520300 encodes phytohormone-related protein of cytochrome P450 family, whose transcriptional level was significantly increased in *sl-MH-1*; Os01g0963000 and Os01g0967000 were the only two down-regulated redox genes in *sl-MH-1* after inoculation, which encodes cationic peroxidase SPC4 and LSM domain containing protein, respectively. SPC4 potentially involved in ROS generation in rice [20].

Among the other eighteen redox-related DEGs in *sl*-48hpi/MH86-48hpi, all of which were up-regulated in *sl-MH-1* mutant, five genes, Os06g0102100, Os02g0204700, Os06g0680700, Os10g0320100 and Os06g0501900, encode cytochrome P450 family proteins; Os10g0356000 and Os10g0502400 mainly expressed in photosynthetic system, the later was regulated by phytochromes and cryptochromes under different light conditions [21, 22]; Os08g0104600 encodes an chloroplast-located ferredoxin, involved in transferring electron from photosystem I to NADP^+^ and the various acceptor systems of metabolic processes [23]; Os03g0290300 and Os06g0216200 (*OsOPR2*) encodes a ω-3 fatty acid desaturase and a OPR isozyme, respectively, which are involved in JA biosynthesis [24–26]; Os04g0459500 encodes a glyceraldehyde-3-phosphate dehydrogenase (GAPDH), which translocates to the nucleus during apoptosis and influences cytotoxicity [27]. To confirm the above RNA-seq results, we performed quantitative real-time PCR (qRT-PCR) analysis of the key genes in redox pathway, including Os01g0963000, Os08g0104600 and Os03g0290300, in MH86 and *sl-MH-3* before and at 48 h after *P. oryzae* inoculation. As shown in Fig. S6, the transcriptional changes of these genes in *sl-MH-3* are similar with those in *sl-MH-1* transcritome data.

In addition, we investigated *SL* transcription in the *japonica* rice cultivar Nipponbare (NPB) and *Piz-t*-transgenic NPB (NPB-Pizt) plants before and after inoculation with the blast fungal isolate KJ201, which is avirulent to *Piz-t* [19, 28]. As shown in Fig. 7c, no significant difference of *SL* transcriptional levels in NPB and NPB-Pizt was detected before or at 48 h after inoculation. With disease development, *SL* transcription in NPB was significantly up-regulated at 72 hpi, when it was not obviously altered in NPB-Pizt (Fig. 7c). These results suggest that successful *P. oryzae* infection could up-regulate *SL* expression in rice.

## Discussion

### Sekiguchi lesion is resulted from high accumulation of endogenous ROS

The biological functions of serotonin in rice are mainly explored via characterizing phenotypes of the *sekiguchi lesion* mutant, as SL is required for converting tryptamine to serotonin. In this study, we identified the mutants of *SL* gene in MH86 background, which also exhibit sekiguchi lesion under normal growth condition and markedly decreased serotonin content. *sl-MH-1* mutant accumulates high level of H_2_O_2_ at its lesion sites and is more sensitive to exogenous H_2_O_2_ treatment. Furthermore, VC treatment could suppress the lesion formation on *sl-MH-1* leaves, and the similar result has been described in the *sl* mutant [29]. These results suggest that when SL is defective, high endogenous oxidation level leads to cell death occurrence, which is consistent with the role of serotonin as an effective internal ROS scavenger [30]. Meanwhile, when *SL* is mutated, the H_2_O_2_ generated from oxidation of the excessive tryptamine by monoamine oxidase could also contribute to the lesion formation [29]. Excessive ROS production could contribute to or execute PCD by rapidly oxidizing and damaging cellular components, including proteins, nucleic acids and lipids. ROS-induced PCD has been described as a mechanism of photooxidative damage in plants during photosynthesis [31]. It has been reported that the most effective wavelength for inducing typical sekiguchi lesion was 400–700 nm; whereas in the dark, only brown spots and necrotic spot lesions formed on the *sl* leaves [32]. In addition, sekiguchi lesion could be significantly inhibited by the photosynthetic inhibitor [33]. Therefore, we speculate that under normal growth condition, serotonin is essential for scavenging endogenous oxygen radicals produced during photosynthesis, and thus protecting rice from oxidative damage.

### SL negatively regulates rice resistance to biotrophic pathogens

In this study, we found that *sl-MH* mutants displayed enhanced resistance to both blast fungus and blight bacteria. *sl-MH-1* formed the typical lesion surrounding the infection sites, which could efficiently inhibit the pathogen extension. Moreover, the transcriptional level of *SL* was up-regulated in NPB but not NPB-Pizt at 72 hpi with the blast fungal isolate avirulent to *Piz-t*, suggesting that a compatible interaction between rice and *P. oryzae* could induce *SL* expression. It has been supposed that 72 hpi is the stage when *P. oryzae* transits from the biotrophic to the necrotrophic phase during its disease cycle [34]. Therefore, the up-regulation of *SL* expression at this time point may be required for alleviating high oxidative stress caused by host cell collapse upon entering necrotrophic phase. Similarly, serotonin was indicated to function in protecting uninfected tissues from oxidative damage caused by the HR [35].

Our data also showed that *sl-MH-1* mutant displayed more robust PTI responses, including PAMPs-induced ROS burst and callose deposition. High oxidative level in plant cells plays multifaceted signaling roles in mediating the establishment of immune responses [36]. For example, ROS could stimulate a rapid Ca^2+^ influx upon elicitation [37]. All the plant plasma membrane-localized respiratory burst oxydase homologs (Rbohs), which are essential for apoplastic ROS production [38], contain the Ca^2+^ − binding EF hand motifs at their N terminus, implying that ROS production and Ca^2+^ influx co-regulate each other [39]. Except that, ROS directly mediates the cross-linking of plant cell wall components to strength the structural barriers against pathogens [40]. Moreover, ROS could also contribute to the activation of plant immune responses by inducing changes in gene expression [41]. Thus, we suppose that the enhanced PTI responses of the *sl-MH* mutant may also be resulted from its high endogenous ROS accumulation.

In contrast to its enhanced resistance to *P. oryzae* and *Xoo*, the *sl* mutant was reported to be more susceptible to *B. oryzae* [12]. The divergent pathogenic phenotypes on *sl* mutants could be resulted from the different trophic types of these pathogens. *B. oryzae* is a necrotrophic pathogen, which takes nutrition from dead host cells. In this case, the spontaneous cell death of *sl* mutants could be a favorable term for *B. oryzae* growth. However, *P. oryzae* and *Xoo* are hemibiotrophic and biotrophic pathogens, respectively, to whose colonization, lesion of *sl* mutants could set a limitation.

### SL functions in suppressing accumulation of the defense hormones

SA and JA are the important phytohormones that trigger and mediate a series of defense responses. In Arabidopsis, JA is involved in resistance against necrotrophic pathogens and herbivorous insects [42], whereas SA contributes to defense responses against biotrophic pathogens [43]. And the SA and JA-mediated immune pathways in Arabidopsis are always antagonistic to each other. Although the role of SA in defense is conserved in rice, the SA content is less essential for inducing rice resistance, as rice plants usually accumulate high level of SA during normal growth [44, 45]. Interestingly, SA could also contribute to rice defense against brown planthopper [46]. Furthermore, in contrast to the finding in Arabidopsis, JA in rice positively regulates immune responses against (hemi)biotrophic pathogens [47, 48].

Our results indicated that SA constantly accumulates in *sl-MH-1* mutant to a level about twice as high as that in the wild type, and the contents of JA and JA-Ile are dramatically increased in *sl-MH-1* when the lesions appear. Considering that SA in rice is essential for modulating redox balance and scavenging the endogenous ROS [49], we speculate that the high SA accumulation in *sl-MH-1* may be employed to alleviate its internal oxidative stress. JA is a lipid-derived hormone, whose chloroplastic intermediate, cis-(1)-12-oxophytodienoic acid (OPDA), is derived from oxidatively modified polyunsaturated fatty acids [50]. Thus the increased JA and JA-Ile contents in *sl-MH-1* may also be caused by the raised oxidative level, as high levels of JA and its derivant were detected in Arabidopsis when more ROS was accumulated by deletion of Fd2, the major ferredoxin in chloroplasts [23]. On the other hand, Os03g0290300, encoding a ω-3 fatty acid desaturase that is involved in the synthesis of unsaturated fatty acids serving as JA precursor, was significantly up-regulated in *sl-MH* mutants, which could contribute to JA and JA-Ile production as well.

## Conclusions

In this study, we identified *sl* mutants in MH86 background (*sl-MH*) and revealed the roles of SL in rice innate immunity. Our results suggest the following conclusions: (i) the *sl-MH* mutant is more sensitive to exogenous ROS stress, its lesions formation is mainly resulted from excessive accumulation of internal ROS; (ii) SL negatively regulates rice defense against blast fungus and blight bacteria via compromising the PTI responses and suppressing the defense hormones accumulation; (iii) loss function of SL dramatically alters transcription of the genes involved in redox pathway after inoculation with *P. oryzae*; (iv) success *P. oryzae* infection up-regulates the transcriptional level of *SL* in rice. Taken together, our study decipher the negative roles of SL in rice defense against (hemi)biotrophic pathogens.

## Methods

### Plant materials and blast isolates

The *indica* rice restore line Minghui 86 was originally bred at Institute of Rice, Fujian Academy of Agricultural Sciences, Fuzhou, China. The *sl-MH-1* mutant was generated from the tissue culture-induced mutation of MH86 as described previously [15]. The *sl-MH-2* and *sl-MH-3* mutants in this study were generated from ^60^Co ~ γ-ray irradiation of MH86 by us. The rice cultivar Nipponbare, its transgenic line carrying *Piz-t* (NPB-Pizt) and the *P. oryzae* isolate KJ201 were originally obtained from Dr. Guo-Liang Wang’s laboratory (Department of Plant Pathology, Ohio State University, Columbus, Ohio). The *P. oryzae* isolate FJ-1 (virulent to Minghui86) was provided by Dewei Yang (Institute of Rice, Fujian Academy of Agricultural Sciences, Fuzhou, China). The *Xoo* strain race 6 was provided by Dr. Dingzhong Tang (Plant Immunity Center, Fujian Agriculture and Forestry University, Fuzhou, China). For the H_2_O_2_ treatment, *P. oryzae* inoculation, PTI responses detection and defense hormones measurement assays, the plants were germinated and grown in a growth chamber at 28 °C under a 12-h light (600–800 μmol/m^2^.s) /12-h dark cycle. For the metabolites measurement, lesion staining, VC treatment and *Xoo* inoculation assays, the plants were grown in a humidity-controlled greenhouse under natural conditions during summer season.

### Blast fungus and blight bacteria inoculation

*P. oryzae* and *Xoo* inoculations were carried out in growth chamber and greenhouse, respectively. *P. oryzae* isolate FJ-1 was cultured on CMII medium for 2 weeks under light for sporulation. Then a conidial suspension with 3 × 10^5^ spores/ml was sprayed on 3-week-old rice leaves. After inoculation, the seedlings were maintained in the dark for 24 h at 28 °C with high humidity, then transferred into the growth chamber for 5 ~ 7 days to evaluate their disease symptom. Four-week-old rice plants were used to perform punch inoculation as previously described [19], and a 10-μl volume of a spore suspension (2 × 10^5^ spores/ml) was applied. Investigation of the fungal biomass in infected rice leaf tissue was carried out as the method described previously [51]. Two punched leaves from one single plant were collected as one biological replicate for the statistic analysis. For leaf sheath inoculation, conidial suspension was injected to the detached sheath cavum of 21-day-old rice plants. Then, the inoculated sheath was kept in an incubator with 80% humidity for the indicated time. Prior to microscopy observing, the surface cell of inner sheath was peeled and made as a slide sample. Inoculation with *Xoo* was conducted during the tilling stage by the leaf clipping method [52]. The bacterial suspension with optical density at 600 nm (OD_600_) = 0.5 was used to inoculate rice leaves as follows: scissor was dipped in the bacterial suspension and then used to cut the tip of a rice leaf. The inoculated plants were kept in the greenhouse for 14 days before disease symptom analysis. The blight lesion length and bacterial population accumulated in the infected leaves were evaluated as reported previously [53].

### VC treatment

VC (0.1%, diluted with distilled water) was sprayed onto the 3-week-old rice seedlings three times per day (at 9:00, 12:00 and 15:00) for 10 days. Water spraying was used as control.

### Analysis of metabolites content

1000 μl of precooled extract solution (acetonitrile:methanol:water, 2:2:1) was added to each 50 mg ground leave samples of MH86 and sl-MH-1. Metabolites extraction was performed as described previously [54] with minor modification as below. After homogenate-sonicate circles and centrifugation, 100 μl of the supernatant was transferred and dried under a nitrogen flow, then 100 μl of 10% methanol was used to reconstitute the residual. Following centrifugation, 80 μl of the supernatant was transferred into an auto-sampler vial for UHPLC-MS/MS analysis. Preparing the standard solution and the UHPLC separation was carried out as described before [54]. L-2-Chloro-phenylalanine was used as the internal standard (IS) at a concentration of 200 nmol/l. Mobile phase A was 0.1% acetic acid in water, and mobile phase B was methanol. The elution time and gradient for UHPLC separation and quantification analysis of the metabolites content were shown in Table S3. Reproducibility was assessed using five biological replicates in each experiment.

### SA and JA contents measurement

For measuring the defense hormones contents, the leaves from three 6-week-old MH86, *sl-MH-1* or *sl-MH-3* plants were harvested as one biological replicate; the leaves from eight 3-week-old MH86 or *sl-MH-1* plants were harvested as one biological replicate. The method of measurement is as previously described [55].

### DAB and Trypan blue staining

The leaf samples of 8-week-old MH86 and *sl-MH-1* plants were soaked in 2 mg/mL 3,3′-diaminobenzidine (DAB) (2 mg/mL DAB,0.05% Tween 20,10 mM Na_2_HPO_4_) for 4 ~ 5 h then transferred to the destaining solution (ethanol:lactic acid: Glycerol = 3:1:1). After heating to 90–95 °C for 15 min, the leaves were transferred to a fresh destaining solution and shaked gently overnight. Trypan blue staining was carried out as previously described [56].

### Callose deposition

For observing callose deposition, the rice leaves from 7-day-old seedling were treat with chitin (hexa-N-acetylchitohexaose). The assay was carried out as previously described [57]. Finally the leaves were observed under UV light (340 to 380 nm; Zeiss LSM880).

### Seedling treated with H_2_O_2_

The dehusking rice seeds were sterilized with 75% ethanol for 2 min and 3% sodium hypochlorite for 30 min, then germinated on 1/2 MS medium with or without 0.02% H_2_O_2_. The plates were put in a 28 °C growth chamber with a 12-h light/12-h dark cycle for 7 days. Then the seedlings were imaged for analyzing sensitivity to H_2_O_2_ treatment.

### ROS burst detection

Leaf discs from 7-day-old rice seedlings were floated on sterilized water overnight, then put into 100 ul reaction solution (20 uM luminal and 2.5 μg/mL peroxidase) containing 500 nM flg22 or 400 nM chitin to immediately test the ROS burst. The luminescence was measured by Berthold Mithras luminometer every 1–2 min for 1 h. Each data point represented eight replicates.

### RNA-seq sequencing and qRT-PCR analysis

The total RNA from MH86 and *sl-MH-1* leaves before and after blast fungus spraying inoculation was extracted using the RNAeasy kit (Qiagen, Germany) and treated with an RNase-Free DNAse Set (Qiagen, Germany), according to the manufacturer’s instructions. The RNA quality, library construction and size were assayed using a 2100 Bioanalyzer system (Agilent, USA). The libraries were synthesized using the TruSeq RNA Sample Preparation v2 kit (Illumina, USA). Total RNA from each treatment was pooled and then two libraries were constructed and used for sequencing. The samples were run in the NovaSeq system and raw sequences of paired 150-bp were obtained.

The analysis below was run on Majorbio (https://www.i-sanger.com). Raw reads were evaluated using fastx_toolkit_0.0.14. SeqPrep and Sickle software were used for clean data. TopHat2 was used to map reads individually for each biological replicate in MH86 or *sl-MH-1* against *O. sativa japonica* cultivar Nipponbare sequence according to MSU 7.0. Cufflinks was used for assembly into transcripts with reference annotation to guide assembly [58]. All the produres were completed according to the settled parameter. The significant differentially expressed loci (SDEL) in *sl-MH-1* mutant compared to MH86 were identified after applying multiple corrections (FDR adjusted *p* ≤ 0.05). The SDEL with fold change ≥2 or ≤ 0.5 were used for further analysis. Functional enrichment analysis (FEA) was performed on SDEL in *sl-MH-1* compared to MH86. Functional annotation and expression correlation analysis were conducted on the gene set related to oxidation-reduction biological process. Heat map was generated using cluster analysis.

qRT-PCR analysis was carried out as previously described [23]. The primers used for qRT-PCR in this study are listed in Table S4.

## Supplementary Information

**Additional file 1: Fig. S1.** Lesion phenotype of the allelic *sl-MH* mutants The leaves of MH86, *sl-MH-1*, *sl-MH-2* and *sl-MH-3* were photographed after grown in greenhouse for 6 weeks.

**Additional file 2: Fig. S2.** Schematic representation of *SL* gene structure and the mutation sites. Black boxes and lines indicate exons and introns, respectively, and untranslated regions are shown in grey boxes. The arrows indicate the mutation sites of the allelic *sl-MH* mutants.

**Additional file 3: Fig. S3.** The content of L-glutamine in MH86 and *sl-MH-1*. The levels of L-glutamine in 8-week-old MH86 and *sl-MH-1* plants were measured by UPLC. Bars represent mean values ± SD from five biological replicates. Statistically significant difference was indicated by ** (*p* < 0.01, Student’s *t*-test).

**Additional file 4: Fig. S4.** Resistance of *sl-MH-3* to *P. oryzae* and *Xoo*. a. 3-week-old MH86 and *sl-MH-3* seedlings were inoculated with *P. oryzae* conidia by spraying and the diseased leaves were imaged at 7 dpi. b. Punch inoculation with *P. oryzae* conidia was performed on the leaves of 4-week-old MH86 and *sl-MH-3* plants. The diseased leaves were photographed at 9 dpi (left), and the fungal biomass was measured (right). Bars represent mean values ± SD from three biological replicates. Statistically significant difference was indicated by ** (*p* < 0.01, Student’s *t*-test). c. MH86 and *sl-MH-3* plants were inoculated with *Xoo*. The infected leaves from three independent MH86 or *sl-MH-3* plants were imaged at 14 dpi. d. Blight lesion length on *sl-MH-3* and MH86 leaves were measured (left), bars represent mean values ± SD (*n* = 6, from three independent plants; * means *p* < 0.05 by Student’s *t*-test). Blight bacterial populations were counted 14 dpi with bars representing mean values ± SD from three independent MH86 or *sl-MH-3* plants (right), ** means *p* < 0.01 by Student’s *t*-test. e. Callose deposition on MH86 and *sl-MH-3* leaves after chitin treatment was imaged with a microscope under UV light (left). Number of the callose deposition per view was counted (right). Bars represent mean values and SD (*n* = 5). Statistically significant difference was indicated by ** (*p* < 0.01, Student’s *t*-test).

**Additional file 5: Fig. S5.** Contents of defense hormones in 3-week-old MH86 and *sl-MH-1* plants. The resting levels of SA, JA and JA-Ile in 3-week-old MH86 and *sl-MH-1* leaves were measured by UPLC. Bars represent mean values ± SD from three biological replicates. Statistically significant difference was indicated by ** (*p* < 0.01, Student’s *t*-test).

**Additional file 6: Fig. S6.** qRT-PCR analysis of the representative genes of redox pathway in MH86 and *sl-MH-3*. The transcriptional levels of Os01g0963000, Os08g0104600 and Os03g0290300 were determined in MH86 and *sl-MH-3* at 48 hpi with *P. oryzae* (water spraying was employed as mock treatment). *UBQ* was used as the internal control. Bars represent mean values ± SD (*n* = 3). Statistically significant difference was indicated by ** (*p* < 0.01, Student’s *t*-test).

**Additional file 7: Table S1.** Significant differentially expressed genes in *sl-MH-1*-mock/MH86-mock.

**Additional file 8: Table S2.** Significant differentially expressed genes in *sl-MH-1*-48hpi/MH86-48hpi.

**Additional file 9: Table S3.** Quantification of serotonin, tryptamine, L-trytophan and L-glutamine by UHPLC-MS.

**Additional file 10: Table S4.** Sequences of the qRT-PCR primers in this study.

## Data Availability

All data sustaining the results in this study are included in this manuscript or its supplementary information files. The datasets analyzed during the current study are available from the corresponding author on reasonable request. The raw sequencing data reported in this article have been deposited in NCBI SRA database, under accession number PRJNA634690, which are publicly accessible at http://www.ncbi.nlm.nih.gov/bioproject/634690.

## Abbreviations

PCD: Programmed cell death
Vc: Vitamin C
PAMPs: Pathogen-associated molecular patterns
PTI: PAMPs-triggered immunity
ROS: Reactive oxygen species
*P. oryzae*: *Pyricularia oryzae*
*Xoo*: *Xanthomonas oryzae* pv. *Oryzae*
*B*: *oryzae*: *Bipolaris oryzae*
redox: Reduction–oxidation
ETI: Effector-triggered immunity
HR: Hypersensitive response
*sl*: *sekiguchi lesion*
DAB: 3,3-diaminobenzidine tetrahydrochloride
MS: Murashige and Skoog
SA: Salicylic acid
JA: Jasmonic acid
JA-Ile: JA-isoleucine
hpi: Hours post inoculation
SDEL: Significant differentially expressed loci
DEGs: Differentially expressed genes
RNA-Seq: RNA-sequencing
qRT-PCR: quantitative real-time polymerase chain reaction
