# Supplementary figures and images for: Loss function of SL (sekiguchi lesion) in the rice cultivar Minghui 86 leads to enhanced resistance to (hemi)biotrophic pathogens
Source: BMC Plant Biol. 2020 Nov 4;20:507. doi: 10.1186/s12870-020-02724-6 (PMC7640399; doi:10.1186/s12870-020-02724-6)

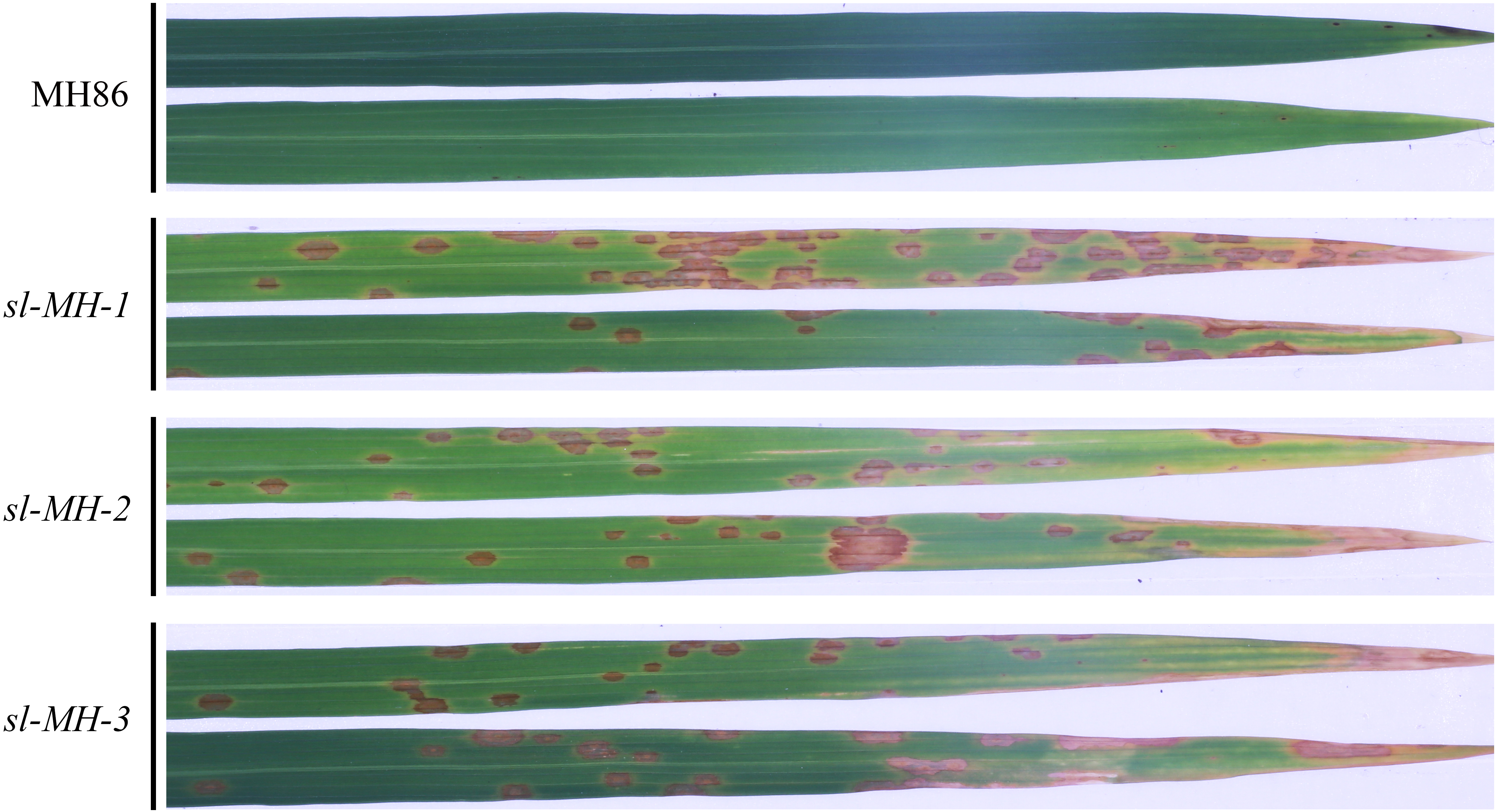

Supplement: Supplementary file 1 — Additional file 1: Fig. S1. Lesion phenotype of the allelic sl-MH mutants The leaves of MH86, sl-MH-1, sl-MH-2 and sl-MH-3 were photographed after grown in greenhouse for 6 weeks. [file 12870_2020_2724_MOESM1_ESM.jpg]

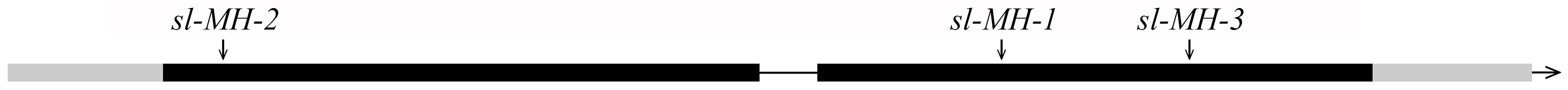

Supplement: Supplementary file 2 — Additional file 2: Fig. S2. Schematic representation of SL gene structure and the mutation sites. Black boxes and lines indicate exons and introns, respectively, and untranslated regions are shown in grey boxes. The arrows indicate the mutation sites of the allelic sl-MH mutants. [file 12870_2020_2724_MOESM2_ESM.jpg]

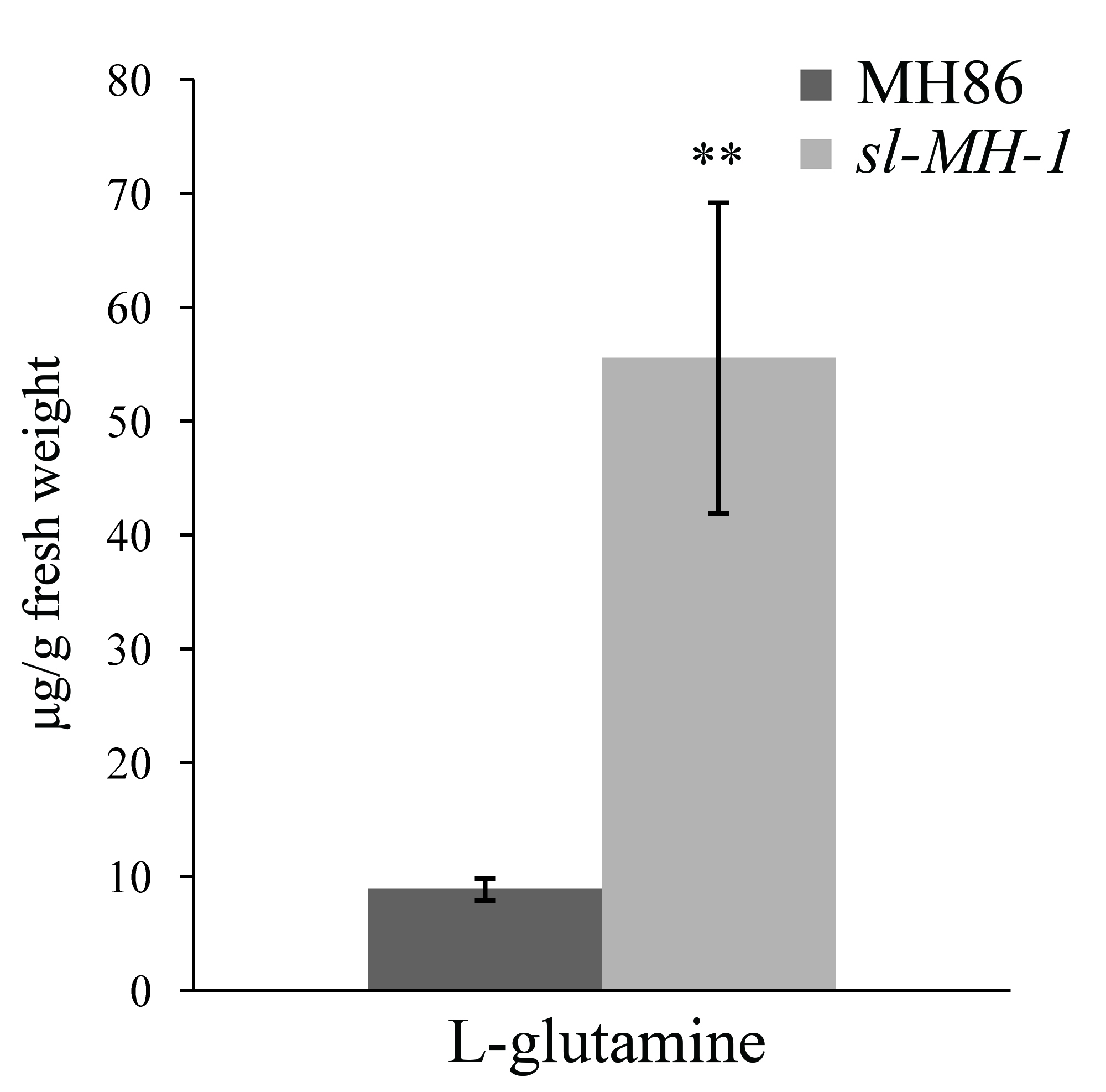

Supplement: Supplementary file 3 — Additional file 3: Fig. S3. The content of L-glutamine in MH86 and sl-MH-1. The levels of L-glutamine in 8-week-old MH86 and sl-MH-1 plants were measured by UPLC. Bars represent mean values ± SD from five biological replicates. Statistically significant difference was indicated by ** (p < 0.01, Student’s t-test). [file 12870_2020_2724_MOESM3_ESM.jpg]

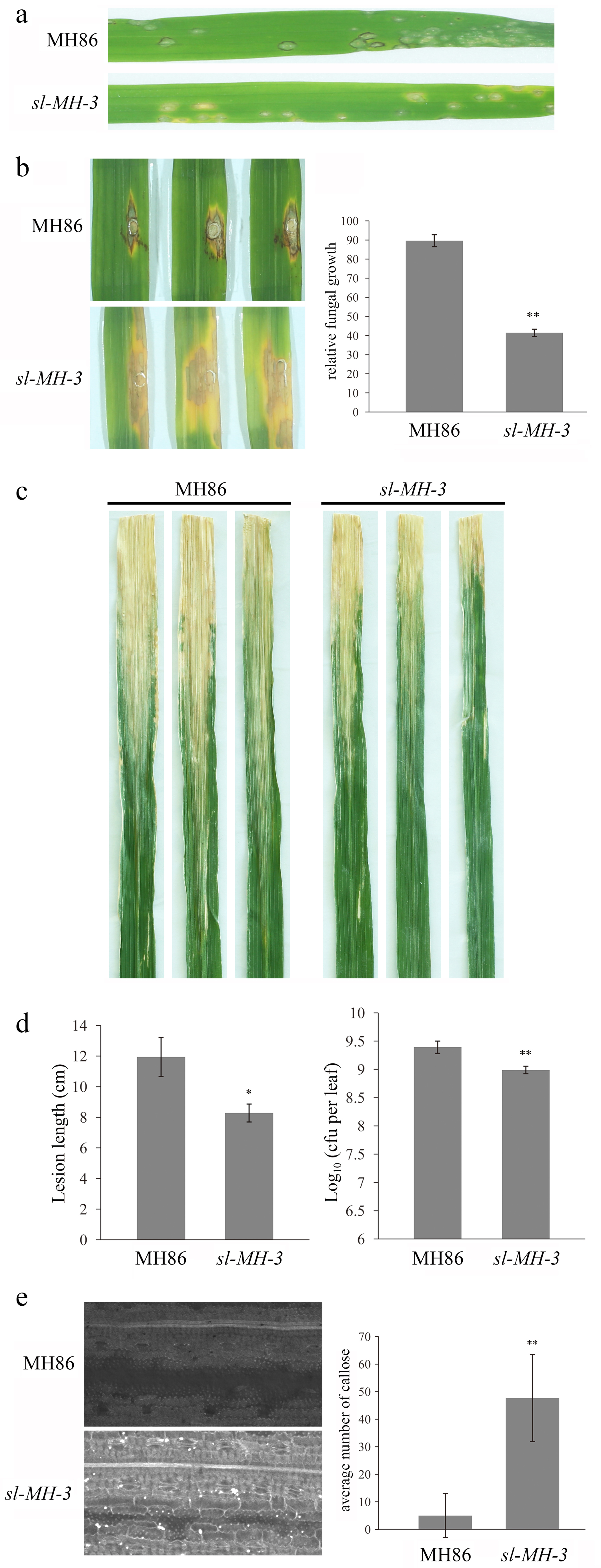

Supplement: Supplementary file 4 — Additional file 4: Fig. S4. Resistance of sl-MH-3 to P. oryzae and Xoo. a. 3-week-old MH86 and sl-MH-3 seedlings were inoculated with P. oryzae conidia by spraying and the diseased leaves were imaged at 7 dpi. b. Punch inoculation with P. oryzae conidia was performed on the leaves of 4-week-old MH86 and sl-MH-3 plants. The diseased leaves were photographed at 9 dpi (left), and the fungal biomass was measured (right). Bars represent mean values ± SD from three biological replicates. Statistically significant difference was indicated by ** (p < 0.01, Student’s t-test). c. MH86 and sl-MH-3 plants were inoculated with Xoo. The infected leaves from three independent MH86 or sl-MH-3 plants were imaged at 14 dpi. d. Blight lesion length on sl-MH-3 and MH86 leaves were measured (left), bars represent mean values ± SD (n = 6, from three independent plants; * means p < 0.05 by Student’s t-test). Blight bacterial populations were counted 14 dpi with bars representing mean values ± SD from three independent MH86 or sl-MH-3 plants (right), ** means p < 0.01 by Student’s t-test. e. Callose deposition on MH86 and sl-MH-3 leaves after chitin treatment was imaged with a microscope under UV light (left). Number of the callose deposition per view was counted (right). Bars represent mean values and SD (n = 5). Statistically significant difference was indicated by ** (p < 0.01, Student’s t-test). [file 12870_2020_2724_MOESM4_ESM.jpg]

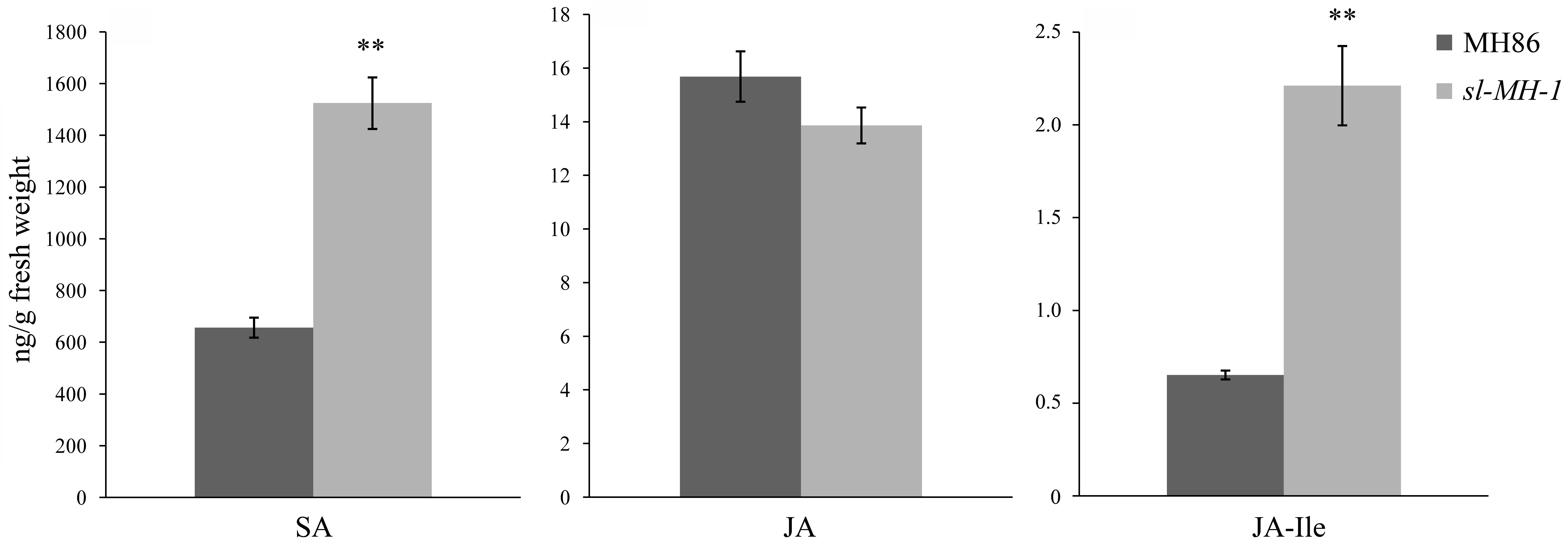

Supplement: Supplementary file 5 — Additional file 5: Fig. S5. Contents of defense hormones in 3-week-old MH86 and sl-MH-1 plants. The resting levels of SA, JA and JA-Ile in 3-week-old MH86 and sl-MH-1 leaves were measured by UPLC. Bars represent mean values ± SD from three biological replicates. Statistically significant difference was indicated by ** (p < 0.01, Student’s t-test). [file 12870_2020_2724_MOESM5_ESM.jpg]

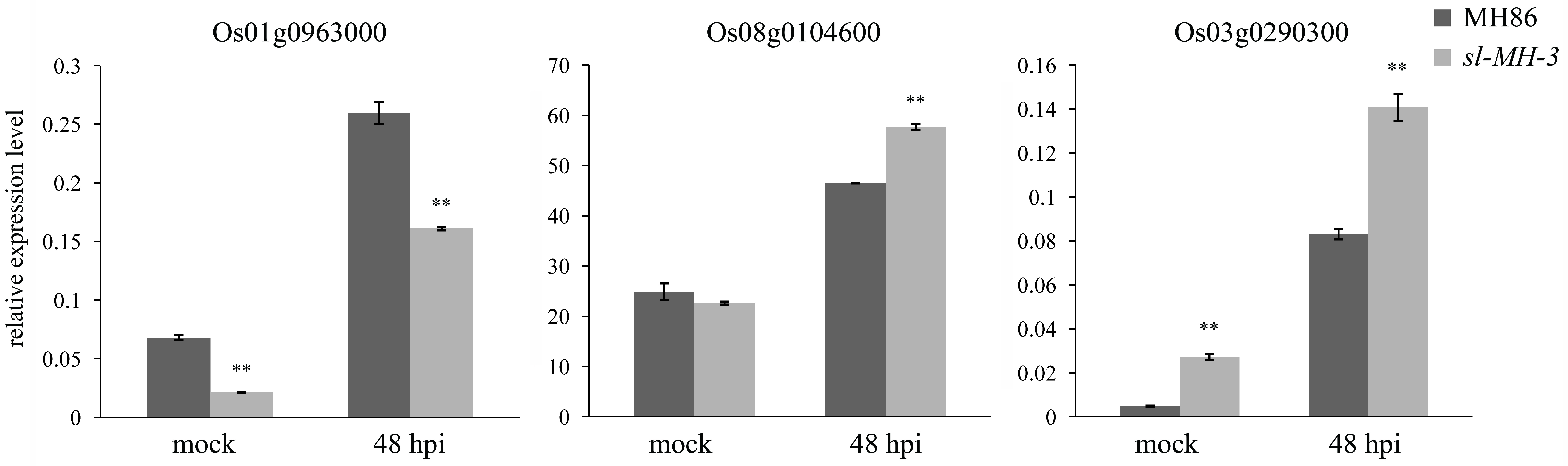

Supplement: Supplementary file 6 — Additional file 6: Fig. S6. qRT-PCR analysis of the representative genes of redox pathway in MH86 and sl-MH-3. The transcriptional levels of Os01g0963000, Os08g0104600 and Os03g0290300 were determined in MH86 and sl-MH-3 at 48 hpi with P. oryzae (water spraying was employed as mock treatment). UBQ was used as the internal control. Bars represent mean values ± SD (n = 3). Statistically significant difference was indicated by ** (p < 0.01, Student’s t-test). [file 12870_2020_2724_MOESM6_ESM.jpg]
